# Playing the field: A retrospective analysis of anterior cruciate ligament injuries and playing position in elite female football (soccer) players

**DOI:** 10.1016/j.jsampl.2026.100159

**Published:** 2026-09-23

**Authors:** Jesse Di Marco, Kylie Fitzgerald, Jane Fitzpatrick

**Affiliations:** a Department of Medical Education, Melbourne Medical School, The University of Melbourne, Australia; b Department of Physiotherapy, Melbourne School of Health Sciences, Faculty of Medicine, Dentistry and Health Sciences, The University of Melbourne, Australia

**Keywords:** ACL, Injury, Football, Soccer, Position, Female

## Abstract

**Background:**

Female football (soccer) athletes occupy different playing positions of goalkeeper, defender, midfielder, and forward that may influence ACL injury risk. This study examined the relationship between ACL injury and playing position in elite female footballers, and analysed player age, injury duration and injury seasonal timing with playing position subgroup analysis.

**Methods:**

Retrospective observational study using third party collected data from public multimedia confirmed elite female football player ACL injuries from 01/01/2022-31/12/2024. Public databases confirmed playing position, age, playing league, injury date and return to competition date. Season injury timing was calculated using league schedule data. De-identified records were analysed; Categorical variables were reported as frequencies and percentages, analysed using Chi–Square tests. Continuous variables were reported as medians and interquartile ranges, analysed using Kruskal–Wallis tests.

**Results:**

From 541 total ACL-injuries, midfielders were most frequently injured (*n* = 183, 33.8%), then defenders (*n* = 169, 31.2%), then forwards (*n* = 149, 27.5%) with no significant difference in frequency of injury (*χ*^2^ = 3.497, df = 2, *p* = 0.174). Goalkeepers were least frequently injured (*n* = 40, 7.4%). ACL injuries were significantly increased in the first (*n* = 74, |Z| = 5.05, *p* < 0.001) and second (*n* = 69, |Z| = 4.27, *p* < 0.001) months of the playing seasons.

**Conclusions:**

All outfield playing positions confer similar ACL injury risk in elite female footballers. The lower goalkeeper ACL injury frequency likely reflects less goalkeepers in the population with reduced cumulative match-play exposure. Playing position injury trends are similar to those observed in elite male football. A high-risk window for ACL injury in the early competitive season is a novel finding for this cohort.

**Key Points**

• Anterior cruciate ligament (ACL) injuries in elite female football (soccer) players result in prolonged absences from competition and long-term consequences.
• Among elite female football players, there is no demonstrated relationship between ACL injuries and playing position.
• The highest frequency of ACL injuries occurs within the first two months of the competitive season.
• A large ACL injury burden signifies the importance of widespread implementation of injury prevention programs.

## 1 Introduction

Anterior Cruciate Ligament (ACL) rupture is a debilitating injury impacting athletes, sporting teams, and health systems worldwide. As of 2023, 16.6 million women and girls play organised football (soccer), 19,064 professionally, with total participation increasing by 25% compared to 2019 [1]. Despite an incidence of 0.6–0.91 injuries/1000 h match exposure [2,3], ACL injuries in female football athletes are highly burdensome relative to other injury types [3]. Generally, ACL injury confers an approximate 4 times increased risk of developing tibiofemoral osteoarthritis [4], and specific sequelae following ACL injury in female footballers include; a 5-fold increased risk of new ACL injury, increased football cessation rates, and decreased general physical activity rates relative to non-injured controls [5].

Football is a team sport with different playing positions. A playing position is defined as the role and special placement of that individual player within an arrangement of players on the playing field. In football, an eleven-player team consists of one goalkeeper and ten outfield players, which are categorised broadly as defenders, midfielders, and attackers. Outfield participation depends on the playing formation a team utilises. Typical formations involve fielding some combination of 3–5 defenders, 2 to 5 midfielders, and 1 to 4 forwards. Importantly, there are different physical exertion characteristics unique to playing positions in female football [6]. This may influence exposure to actions identified as high risk for ACL injury in the literature (e.g. pressing, cutting, regaining balance after kicking, tackling) [7,8], with a predominance of non-contact mechanisms [9,10]. Therefore, we hypothesise playing position may influence ACL injury rates in elite female football, as has been previously observed in collegiate female rugby [11].

Evidence suggests that multicomponent exercise-based injury prevention programs (such as FIFA 11+) reduce ACL injury risk in female footballers by 45% [12]. Therefore, identification of athletes at high risk for ACL injury remains a key priority of research that may assist clinicians to individually target injury prevention and load-management interventions to ensure continuation of participation. Despite the 2–3 times greater risk of ACL injury in female soccer players relative to male soccer players [13], studies to date specifically assessing ACL-injury with reference to playing position in elite football players have been exclusively in male cohorts [[14], [15], [16], [17]]. Studies in elite female cohorts have explored playing position as a risk-factor for general injury, with limited ACL injury data that impedes subgroup analysis by playing position [[18], [19], [20], [21], [22]].

The primary aim of this study was to explore the relationship between ACL injury and playing position in elite female football players, hypothesising that some playing positions confer an increased risk of ACL injury relative to others. Secondary aims of this study included describing trends in the age, return to play (RTP) time, and seasonal timing of injury in ACL injured elite female football players, and to identify whether these parameters are influenced by playing position subgroup analysis.

## 2 Methods

### 2.1 Setting

Deidentified records from the ACL Women’s Football Club (ACLW-FC) dataset were obtained on 08 February 2025 in excel spreadsheet format. The following parameters were included: Playing position, age, injury month, injury year, RTP time (months), and playing league. A multi-source, corroborative approach improved the accuracy of media-derived data. Data collection occurred via social media and public football databases [Soccerdonna (https://www.soccerdonna.de/en/), Soccerway-Women (https://au.women.soccerway.com/), PlayMakerStats (https://www.playmakerstats.com/competitions/football/women)]. These databases utilise a combination of automated data feeds and volunteer-driven crowdsourcing to collate female soccer matchday and individual player statistics that inform player scouting and transfer market valuations. Data collection occurred between March 2023 and December 2024, where social media and Soccerdonna (https://www.soccerdonna.de/en/) were used to identify elite female footballers who experienced ACL injury between 01 January 2022 and 31 December 2024. A three-year period was assessed to increase the observation of ACL injuries for analysis.

### 2.2 Participants

Eligibility criteria defined female football players as ‘elite’ by participation in a first-division national league team or a national team at the time of injury. ACL injury was defined as an ACL injury reported by club/football association/national team media. Specifications of injury severity (e.g. complete/partial tear) and management strategy (e.g. surgical/nonoperative management) were not possible due to the secondary data sampling method. Social media keyword searching in English and Latin-derived languages identified club, football organisation, or national team statements confirming player ACL injury. For ‘cruciate ligament injuries’ identified via Soccerdonna (https://www.soccerdonna.de/en/), social media searching confirmed ACL injury. The data-sourcing method was consistent throughout and records were analysed in de-identified format to reduce bias.

### 2.3 Variables

Playing positions were defined as goalkeeper, defender, midfielder and forward as reported in the Soccerdonna (https://www.soccerdonna.de/en/) and Soccerway-Women (https://au.women.soccerway.com/) databases. Soccerdonna (https://www.soccerdonna.de/en/) was primarily used to allocate playing position. Where the player’s club/league were not listed on this database, Soccerway-Women (https://au.women.soccerway.com/), then PlayMakerStats (https://www.playmakerstats.com/competitions/football/women) playing position allocation was instead used. The same database availability priority was used to confirm player age, playing league, and RTP time. Player age was defined as the age of the player (years) at the time of injury. Playing league was defined as the domestic league in which a player’s team participated in at the time of injury. RTP time was defined as the length of time (months) from date of recorded injury to date of return to official match play. Dates of matches in which injury or return-to-play occurred were derived from database data. Where injury date was not publicly available (e.g. injury in training), date of injury announcement instead was used. Players without a RTP date (players in recovery or missing data) prior to 31 December 2024 were excluded from RTP analysis.

For the relevant playing leagues, the starting month for seasons included in the period of study were searched. The starting month for a season (Month = 1) was defined as the month in which the first competitive game of the domestic playing season occurred. In this model, the month in which the first match of the following season occurred marked the end of the current season and the start of a new season. For each player, the starting month of the season in which injury occurred was coded with the calendar month of injury to determine the month of the playing season in which injury occurred.

### 2.4 Statistical analysis

IBM SPSS Statistics 29.0 was used for all calculations. Categorical variables were summarised using frequencies and percentages and analysed using Chi–Square testing. For significant results, post hoc standardised residuals were calculated. Continuous variables were sub-grouped by playing position and analysed for normality using Q–Q plots, histograms, and Shapiro–Wilk tests. Normality assumptions were violated for all analysed continuous parameters. Therefore, continuous parameters were reported by median and interquartile range (IQR) and analysed by playing position using Kruskal–Wallis tests. Descriptive statistics were tabulated for total ACL injuries, injury year, median age, and median RTP time grouped by playing position. Categorical variables were visualised using bar charts, and continuous variables were visualised using box plots.

## 3 Results

Over the three-year data collection period, 541 ACL injuries were identified (180.33 injuries/year) (Table 1). Injuries from 42 leagues across the six Federation Internationale de Football Association confederations were identified. Injuries were listed in descending order of frequency: Union of European Football Associations (UEFA) (*n* = 321), Asian Football Confederation (*n* = 81), South American Football Confederation (*n* = 71), Confederation of North, Central America and Caribbean Association Football (*n* = 65), Confederation of African Football (*n* = 2), and Oceania Football Confederation (*n* = 1). Within UEFA, 220 ACL injuries were captured from the first division leagues of UEFA’s top 8 women’s associations of France, England, Spain, Germany, Italy, Portugal, Sweden and Norway [23] during the study period. RTP data were available for 285 players, of which 277 players returned to play. Eight players were recorded to retire following ACL injury.

**Table 1:** Total ACL injury characteristics by playing position.

Variable	Goalkeeper	Defender	Midfielder	Forward	Total
**Total injuries**					
Frequency	40	169	183	149	541
Percentage (%)	7.4	31.2	33.8	27.5	100.0
**Injuries by year**					
2022	16	47	54	50	167
2023	10	55	56	56	177
2024	14	67	73	43	197
**Age (years)**^a^	24 ± 7	24 ± 6	24 ± 7	23 ± 6	23 ± 6
**Return to play time**^a^^b^**(months)**	12 ± 4	11 ± 3	11 ± 3	12 ± 4	11 ± 3

a Age and return to play time are expressed as a median ± interquartile range.

b For the *n* = 277 players with available return to injury data.

### 3.1 Playing position

Amongst the outfield playing positions, ACL injuries occurred most frequently in midfielders (*n* = 183, 33.8%), followed by defenders (*n* = 169, 31.2%), then forwards (*n* = 149, 27.5%). The lowest number of ACL injuries was observed in goalkeepers (*n* = 40, 7.4%) (Fig. 1). When goalkeepers were excluded from analysis, a Chi–Square goodness of fit test showed no significant difference between the outfield playing positions of defender, midfielder, and forward compared to expected equal proportions (*χ*^2^ = 3.497, df = 2, *p* = 0.174). When analysing ACL injury distribution by playing position with respect to year of injury, a Chi–Square test of independence detected no significant variation in the ratio of ACL injuries by playing position across the three individual years of study (*χ*^2^ = 7.559, df = 6, *p* = 0.272).

**Fig. 1:**
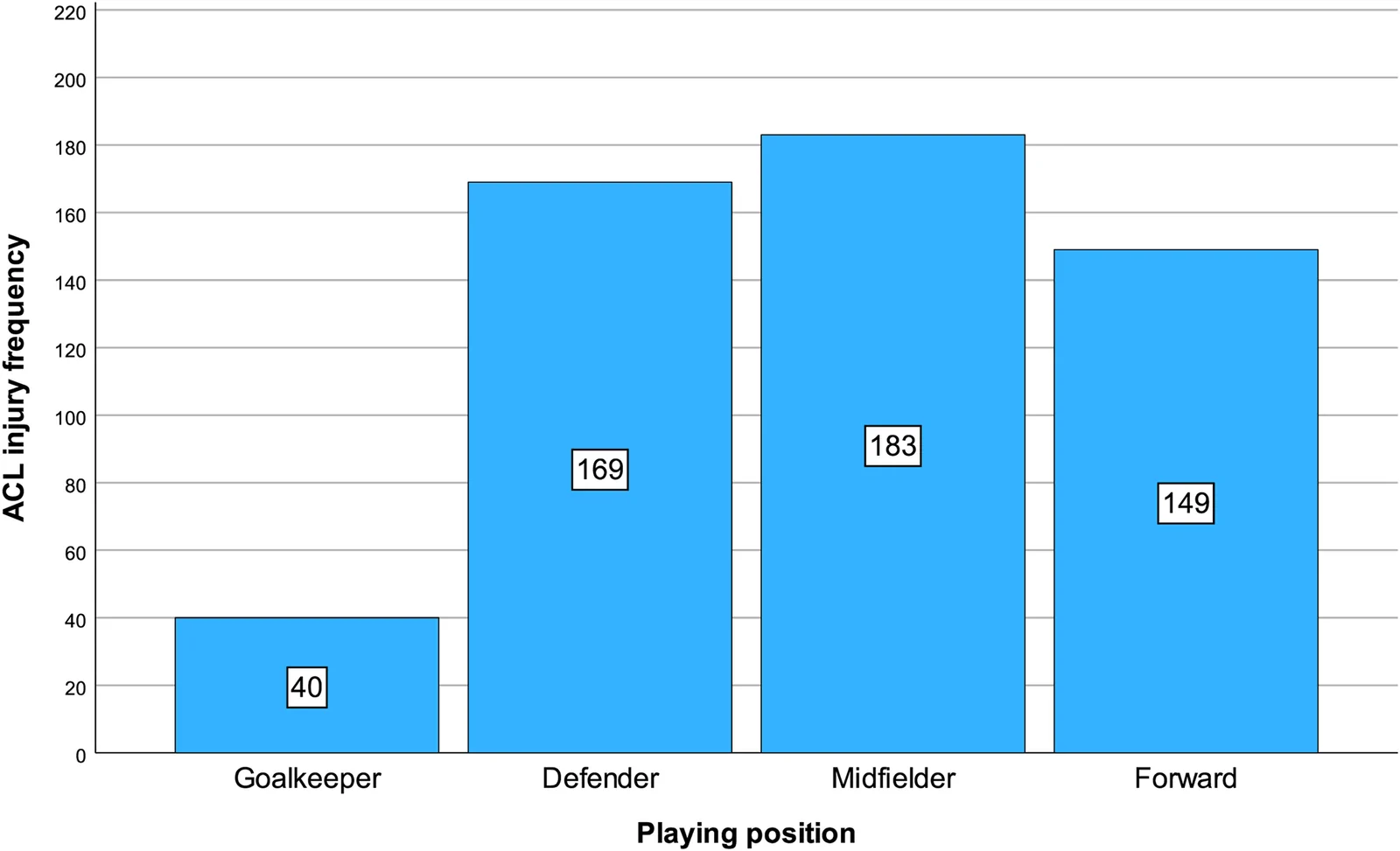
Total ACL injury frequency grouped by playing position.

### 3.2 Age

The median age (±IQR) of ACL injured players was 23 ± 6 years. A Kruskal–Wallis test showed no significant difference in player age across the playing position groups [H(3) = 5.760, *p* = 0.124]. Median age grouped by playing position was as follows: Goalkeepers 24 ± 7 years, Defenders 24 ± 6 years, Midfielders 24 ± 7 years, and Forwards 23 ± 6 years (Fig. 2).

**Fig. 2:**
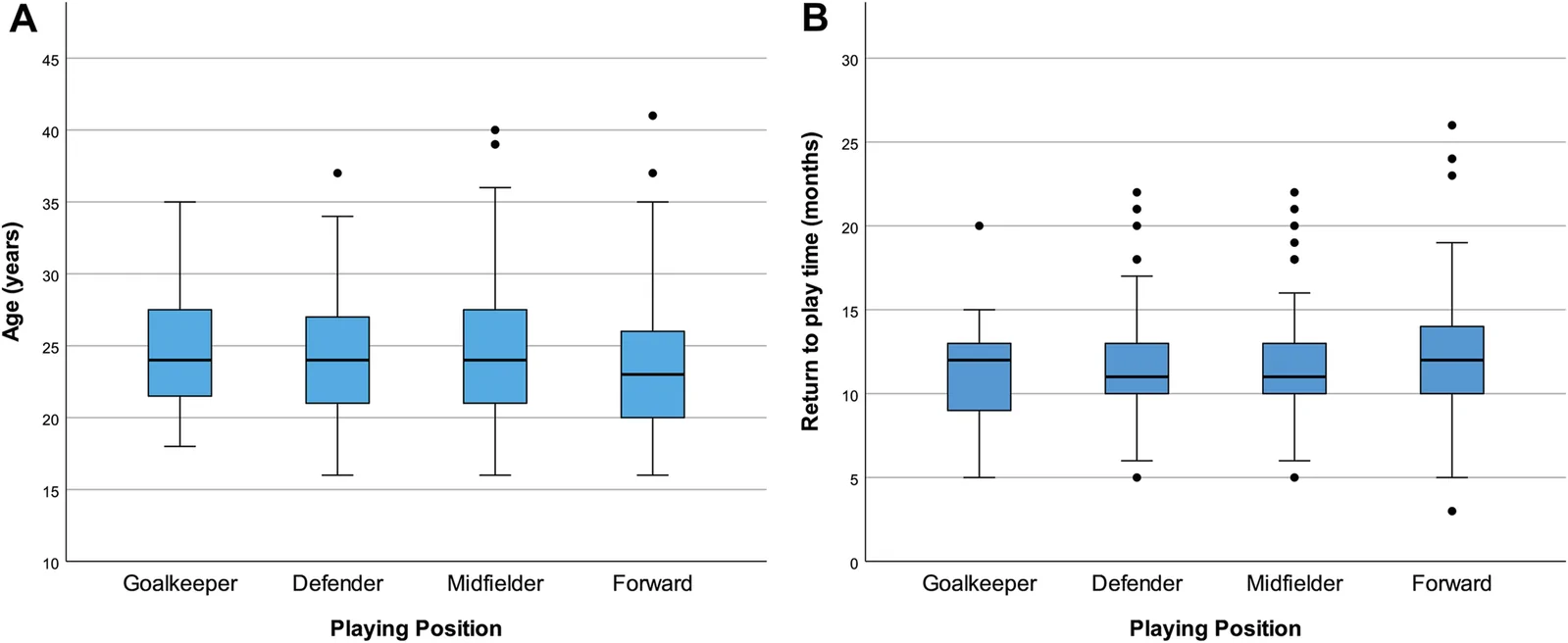
Player age and ACL injury return to play time grouped by playing position. (A) Boxplot showing ACL-injured player age (in years) grouped by playing position. (B) Boxplot showing return to play time (in months) for players following ACL injury grouped by playing position.

### 3.3 Injury duration

The data demonstrated that 277 players returned to match play in a median time of 11 ± 3 months. A Kruskal–Wallis test showed no significant difference in ACL injury duration across the playing position groups [H(3) = 1.607, *p* = 0.658]. Median injury duration grouped by playing position was found to be 12 ± 4 months for both Goalkeepers and Forwards, and 11 ± 3 months for both Defenders and Midfielders (Fig. 2).

### 3.4 Seasonal timing

When analysing frequency of ACL injury by calendar month, a Chi-square goodness of fit test was significant compared to expected equal proportions (*χ*^2^ = 48.908, df = 11, *p* < 0.001). Increased relative ACL injury frequency was observed in March (*n* = 68, |Z| = 3.41, *p* < 0.001) and May (*n* = 59, |Z| = 2.07, *p* < 0.05), whilst decreased relative ACL injury frequency was observed in June (*n* = 25, |Z| = 2.99, *p* < 0.01) and December (*n* = 24, |Z| = 3.14, *p* < 0.01) (Fig. 3).

**Fig. 3:**
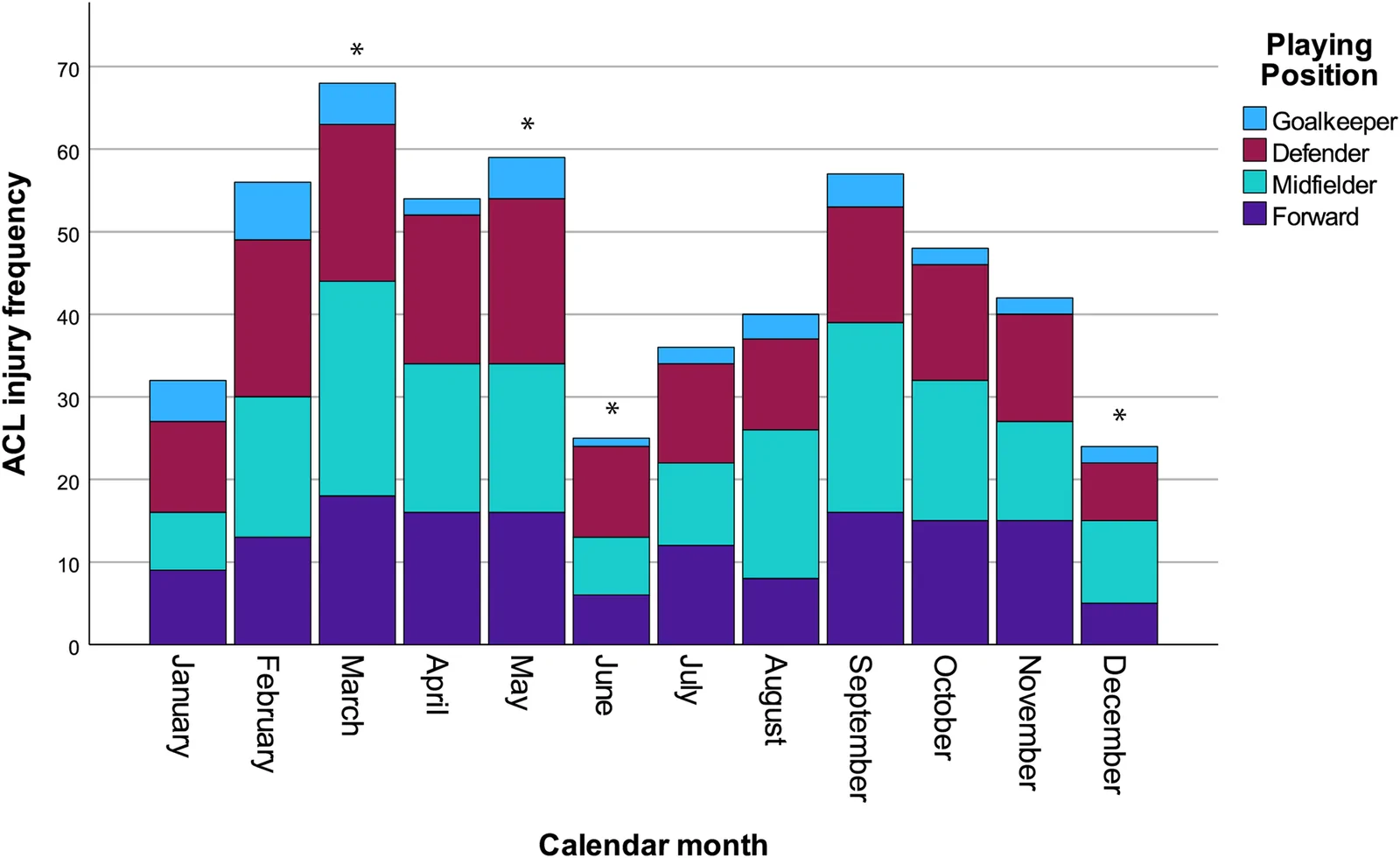
ACL injury frequency tallied by calendar month of injury, stacked by playing position. Note. ∗ Denotes statistically significant values.

When injury month and playing league data were recoded to analyse ACL injury frequency by numerical month of the playing season, a Chi-square goodness of fit test was significant compared to expected equal proportions (*χ*^2^ = 82.844, df = 12, *p* < 0.001). A significantly increased number of injuries were observed in the first month (*n* = 74, |Z| = 5.05, *p* < 0.001) and second month (*n* = 69, |Z| = 4.27, *p* < 0.001) of the playing season (Fig. 4). Additionally, a significantly reduced number of ACL injuries were observed in the 12th month (*n* = 21, |Z| = 3.58, *p* < 0.001) of the season.

**Fig. 4:**
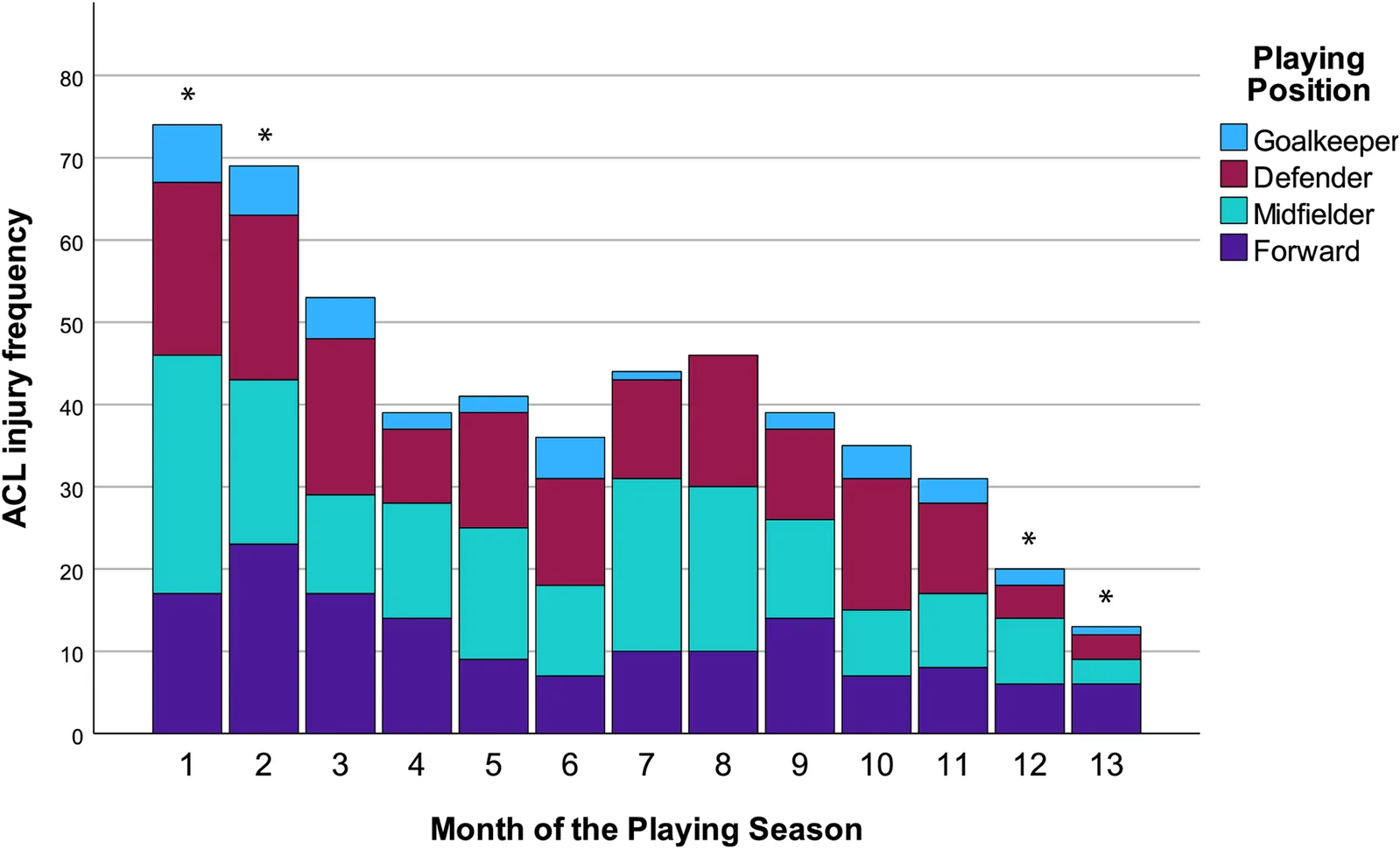
ACL injury frequency tallied by month of the playing season, stacked by playing position Note. ∗ Denotes statistically significant values. Month = 1 is defined as the month in which the first competitive match of a domestic playing season took place. Season *N* can exceed 12 months if the first competitive match of season *N+1* occurred in a later calendar month than the first competitive match of season *N*.

## 4 Discussion

Contrary to hypothesis, this study found no evidence suggesting that particular playing positions confer increased relative ACL injury risk. ACL injury frequency was relatively equal amongst outfield playing positions and comparatively reduced for goalkeepers. Notably, ACL injury frequency was significantly increased in certain months of the year, which corresponded to the early phase of the competitive domestic league season.

### 4.1 Playing position

Analysis suggested that all outfield playing positions were at relatively equal risk for ACL injury. ACL injury frequency was concordant amongst outfield playing position groups. Though non-significant, a slightly reduced ACL injury frequency in forwards relative to midfielders and defenders largely resembles the expected distribution of outfield players and their participation rates in a typical elite squad. Similarly, whilst goalkeeper ACL injury frequency was reduced compared to other positions, one goalkeeper is fielded per ten outfield players. Hence, the reduced ACL injury frequency observed in goalkeepers likely reflects 1) a reduced number of goalkeepers relative to other playing position subgroups in the elite female soccer player population, and 2) a reduced cumulative match-play exposure for goalkeepers relative to other position subgroups. The consistent proportions of ACL injuries in each position across the three years of study suggests that injury trends between playing positions are stable.

General injury studies with playing position subgroup analysis in elite female football cohorts to date have observed limited ACL injuries (2–11 injuries/study) due to sample size (*n* = 41–269) and exposure time (1–2 seasons), preventing meaningful subgroup analysis by playing position [18,19,21,22,24,25]. Additionally, raw data describing ACL injury frequency by playing position is rarely available in the literature for elite female footballers. Only one study [19] tallied ACL injuries by playing position, categorising ten players with 11 ACL-injuries (one defender with ipsilateral re-rupture) as two goalkeepers, three defenders, four midfielders, and one forward.

By contrast, raw data grouped by playing position is available from elite male football studies specifically examining ACL injuries [[14], [15], [16], [17]]. The distribution of ACL injuries by playing position in this study [*n* = 541 ACL injuries, 7.4% goalkeepers, 31.2% defenders, 33.8% midfielders, 27.5% forwards] is consistent with observations in the literature from different elite male populations [*n* = 84–183 ACL injuries, 1.4%–10% goalkeepers, 27.8%–43% defenders, 29.1%–38.9% midfielders, 20%–32% forwards]. Observation of a reduced number of injuries to goalkeepers relative to each outfield playing position is a unanimous finding in elite male cohorts consistent with this study.

### 4.2 Return to play time

RTP findings were consistent with the literature for elite female footballers. The observed median RTP time of 11 ± 3 months (*n* = 277) in this study is consistent with a median RTP time of 12.1 months (IQR 10.9–14.3 months) retrospectively identified from 30 ACL-injured National Women’s Soccer League players (*n* = 30, 2013–2020) [26]. These durations slightly exceed the median duration of 292 days lost per ACL injury (IQR 246–334 days) prospectively identified in the UEFA Women’s Elite Club Injury Study (*n* = 33 injuries, 2018–2022) [3]. Given that ‘days lost’ measured injury duration endpoint as returning to full training or competition, a slightly shortened ‘days lost’ versus RTP time is reasonable considering athletes likely return to training prior to competitive gameplay. This difference may also result from limitations of injury duration calculation used in this study. There was some variation in injury starting point accuracy for cases not reported to occur in a specific game. Additionally, competitive gameplay as an endpoint may over-estimate injury duration; a player may complete recovery in the off-season, delaying return to play. These limitations unlikely affected certain playing positions disproportionately. Thus, the finding of no difference in RTP time by playing position in this study appears robust. This finding has been previously observed in elite female football [26] and is consistently observed in elite male football studies [17,27].

### 4.3 Seasonal timing of ACL injury

This study is the first to report monthly ACL injury data in elite female football players, notably finding an increased ACL injury frequency in the first two months of the playing season.

By calendar month, the distribution of ACL injury frequency appears bimodal, with relatively increased frequency between February–May and September–October. These temporally coincide with the starting months of the 42 different playing league-seasons that occurred during the study period, which primarily occurred in February–May and August–October depending on hemisphere location and competition schedule.

In this study, a significant peak in ACL injury frequency was observed in the first two months of the competitive season, suggesting that ACL injury risk is elevated in the early competitive season period. This finding has been observed previously in elite male football cohorts [15,16,28], National Collegiate Athletic Association soccer, basketball, and lacrosse athletes (*n* = 529 injuries, 2004–2016). [29], and is recently suggested in analysis of Catalan Soccer Federation participants (under-6 youth through to adults, amateur to semi-professional level, *n* = 3381 injuries, 2016–2022). This suggests that a high-risk period for ACL injury in the early season may be generalisable by sex, age, and participation level in football, and may occur across team ball sports more generally.

Rationales in the literature for increased ACL injury risk during the early season relate to playing surface quality and preseason loading. Where the early season coincided with a drier clime, association to dry, firm playing surfaces was considered [15]. Rationales regarding load management in the preseason are mixed; deficits in physical preparedness [28] and enduring adverse effects of preseason conditioning [16] are both considered. Fixture congestion and international travel may also be relevant; recent literature studying 143 professional female footballers found that ACL-injured players had more match appearances, less rest time and travelled more compared to the control group [30]. Thus, further assessment factors that may influence ACL injury risk during the early season, particularly the role of preseason training load given non-consensus in the literature, should be a key priority of future research.

This study observed a significantly reduced number of ACL injuries in month 12 and 13 of the playing season, likely representing the offseason/preseason. This may reflect reduced ACL injury risk with training exposure relative to match exposure in elite female footballers [3]. However, this result should be interpreted cautiously; these months may contain disproportionately reduced ACL injury frequencies due to inclusion of some playing seasons less than 12 months in duration (where the starting month of the following season occurred earlier than the current season).

### 4.4 Limitations

As this retrospective, observational study utilised data that does not inform total population size or player-exposure, ACL injuries were reported by frequency instead of incidence. Analysis of public domain secondary injury data limits accuracy relative to medical personnel injury reporting. Global inclusion improves generalisability to the elite female population, but the elite focus limits generalisability to broader levels of play. Within the elite population, there is risk of under-representing ACL-injuries not reported/searchable, and potential selection bias with non-random selection of players more conducive to public searchability. Calculations of injury timing during the playing season did not account for injuries occurring on international duty. As some players can participate in multiple playing positions in a season, there may be instances where the playing position occupied at time of injury was not the player’s listed position.

### 4.5 Strengths and directions for future research

Despite conferring significant burden, ACL injury is relatively infrequent in elite female footballers [3]. The retrospective approach facilitated analysis of many ACL injuries, providing a novel examination of playing position as an ACL injury risk factor and revealing a high-risk window for injury not previously demonstrated in this population. Future research should prioritise the characterisation of early season ACL injury risk factors. Additionally, future studies incorporating playing position should consider the following: Documentation of specific playing position at the time of injury, prospective tracking of playing position-exposure to account for players occupying multiple roles, and subdivision of outfield position roles into central and wide variations (e.g. defenders into centre backs and fullbacks).

### 4.6 Clinical implications

This study found that seasonal timing rather than playing position was associated with ACL injury frequency, adding to the discourse of static and dynamic ACL injury risk. Epidemiological assessment of ACL injury risk factors is essential to optimize player management and maximize the benefits of preventative intervention. Clinicians can anticipate that RTP timelines are not influenced by playing position. As this study does not reveal a particular subgroup of elite football players at elevated risk of ACL injury, injury prevention programs should be implemented universally in this population.

## 5 Conclusion

This study found that seasonal timing rather than playing position was associated with ACL injury frequency in elite female football players. ACL injury frequency was consistent across all outfields playing position groups. A reduced ACL injury frequency in goalkeepers likely reflects fewer goalkeepers in the population with reduced cumulative match-play exposure. Playing position injury trends are similar to those observed in elite male football. A high-risk window for ACL injury in the first two months of the competitive season is a novel finding in this cohort. Future research should characterise factors that increase ACL injury risk during this critical period to reduce the burden of ACL injuries in elite female football players.

## Contributors

All authors contributed to the study conception and design. Data was collected by VC. Analyses were completed by JD. Results were interpreted by all authors. JD wrote the first draft of the manuscript, which was subsequently revised for important intellectual content by KF and JF. All authors read and approved the final manuscript prior to submission. KF is the guarantor.

## Ethics approval

This project was approved by The University of Melbourne Human Research Ethics committee (approval number 2024-30809-60781-3).

## Ethics declaration

The authors have NOT obtained informed consent from participants or their legal representatives. The authors have chosen a waiver of consent because the data is publicly available, and the impracticability of obtaining individual consent would hinder the research objectives. A waiver of consent is justified as the research involves minimal risk, the data is public, consent is impracticable to obtain, and no adverse effect on participants’ rights or privacy is anticipated. The research has the potential to provide significant benefits to the welfare of participants and the wider community.

This study was performed in compliance with relevant laws, regulatory frameworks and guidelines where the research took place. This study was approved by the University of Melbourne Human Research Ethics committee. (Approval No. 2024-30809-60781-3)

## Funding

None declared.

## Declaration of competing interest

The authors declare that they have no known competing financial interests or personal relationships that could have appeared to influence the work reported in this paper.
